# Supplementary material for: Impact of Endocrine-Disrupting Chemicals in Breast Milk on Postpartum Depression in Korean Mothers
Source: Int J Environ Res Public Health. 2021 Apr 22;18(9):4444. doi: 10.3390/ijerph18094444 (PMC8122652; doi:10.3390/ijerph18094444)
Supplement: Supplementary file 1 [file ijerph-18-04444-s001.zip › ijerph-1194820-supplementary.pdf]

Supplementary table S1. Spearmann correlation among phthalates, BPA, triclosan, and paraben in breast milk

Chemicals	MEP	MnBP	MiBP	MEHP	MiNP	MBzP	BPA	TCS	MP	EP	PP
MEP	1.000	0.919**	0.630**	0.016	0.021	0.116	0.029	-0.030	0.026	-0.082	-0.010
MnBP		1.000	0.663**	0.142*	0.138*	0.257**	0.086	-0.034	0.109	0.006	0.076
MiBP			1.000	0.356**	0.684**	0.445**	0.262**	-0.030	0.748**	0.274**	0.682**
MEHP				1.000	0.637**	0.382**	0.205**	-0.027	0.554**	0.233**	0.589**
MiNP					1.000	0.540**	0.343**	-0.019	0.947**	0.405**	0.927**
MBzP						1.000	0.262**	-0.023	0.554**	0.292**	0.520**
BPA							1.000	0.277**	0.371**	0.399**	0.476**
TCS								1.000	0.000	0.139*	0.098
MP									1.000	0.444**	0.959**
EP										1.000	0.499**
PP											1.000

MEP, mono ethyl phthalate; MnBP, mono-N-butyl phthalate; MiBP, mono-isobutyl phthalate; MBzP, monobenzyl phthalate; MiNP, mono-isononyl phthalate; MEHP, mono (2-ethylhexyl) phthalate; BPA, bisphenol A; TCS, triclosan; MP, methyl paraben; EP, ethyl paraben; PP, propyl paraben

* $p < 0.05$, ** $p < 0.01$
